# New molecular markers to differentiate carbon dioxide intoxication from asphyxia due to oxygen deficiency

**DOI:** 10.1007/s12024-025-00981-1

**Published:** 2025-04-01

**Authors:** Masahiko Yatsushiro, Midori Katsuyama, Takuma Nakamae, Kotomi Imahara, Machiko Miyamoto, Takahito Hayashi

**Affiliations:** Department of Legal Medicine, Graduate School of Medical and Dental Sciences, Kagoshima University, 8-35-1 Sakuragaoka, Kagoshima, 890-8544 Japan

**Keywords:** Carbon dioxide intoxication, Asphyxia due to oxygen deficiency, Molecular markers, Postmortem differential diagnosis

## Abstract

**Purpose:**

The lack of specific autopsy findings for carbon dioxide (CO_2_) intoxication hinders the determination of cause of death based on autopsy findings alone. In addition, when death occurs in a space is filled with CO_2_ or other gases, the cause of death must be distinguished between intoxication and asphyxia due to oxygen deficiency, which also has no specific autopsy findings. In this study, we aimed to identify diagnostic markers of mRNA expression in the brainstem that indicate cause of death in cases of suspected CO_2_ intoxication.

**Methods:**

Mouse models of CO_2_ intoxication (composition of ambient gases at 70% CO_2_, 20% O_2_, and 10% N_2_) and asphyxia due to oxygen deficiency (5% O_2_, 95% N_2_) were used to identify mRNA markers specific to intoxication or asphyxia.

**Results:**

Using RNA-Sequence analysis, we identified 7 candidate genes for qRT-PCR analysis: Acid-sensing ion channel 4 (Asic4), Early growth response protein 1 (Egr1), Neurogranin (Nrgn), Opioid receptor delta 1 (Oprd1), Semaphorin 3f (Sema3f), Transthyretin (Ttr), and Tryptophan hydroxylase 2 (Tph2). We observed a significant increase of Nrgn mRNA expression in the brainstem of CO_2_ intoxication and a significant increase of Ttr mRNA expression in the brainstem of asphyxia due to oxygen deficiency.

**Conclusion:**

Assays for the expression of Nrgn and Ttr in the human brainstem may assist in the diagnosis/differential diagnosis of CO_2_ intoxication and asphyxia due to oxygen deficiency, respectively.

## Introduction

Fatalities due to exposure to high concentrations of carbon dioxide (CO_2_) have been reported occasionally from industrial accidents in the brewing industry, the refrigeration and freezing industry, and fire extinguishing equipment inspection work [1–4]. In addition, in rare cases, death may occur due to CO_2_ being generated from dry ice or a mixture of baking soda and citric acid for the purpose of suicide in Japan [5–7]. The accurate pathophysiology of CO_2_ intoxication is not known, but the following mechanisms have been assumed. Inhalation of extremely high concentrations of CO_2_ causes a rapid increase in the rate at which CO_2_ diffuses and permeates the alveolar walls and dissolves into the blood and tissue fluids in proportion to the partial pressure difference, resulting in accumulation of CO_2_ in the body and subsequent respiratory acidosis. In respiratory acidosis, K^+^ is released extracellularly in exchange for H^+^, which leads to an elevation in blood K^+^ levels, resulting in lethal arrhythmias and death, or respiratory arrest in a short time due to the suppression of the respiratory center in the medulla oblongata by hypercarbonemia [2, 8]. In any of the above pathophysiological mechanisms, the patient dies within the short time before morphological and histological changes occur, and forensic autopsies of such cases show no specific findings [2–4, 6–8]. Therefore, it is difficult to diagnose CO_2_ intoxication based on autopsy findings alone. In such cases, diagnosis is made either by full-scale replication experiments at the scene or by simple laboratory experiments using a scaled model to confirm whether or not CO_2_ is generated in lethal concentrations [7]. These methods are expensive and time-consuming and may not prove definitive answers. Thus, the development of molecular markers capable of diagnosing CO_2_ intoxication postmortem is needed.

Furthermore, if an enclosed space is filled with a gas (e.g., helium gas) other than oxygen (O_2_), the partial pressure of O_2_ in that space will be significantly reduced, and death by asphyxia due to oxygen deficiency should be considered [9, 10]. Therefore, in such cases, it is important to make a differential diagnosis as to whether the death is due to CO_2_ intoxication or asphyxia due to oxygen deficiency. However, in the case of acute asphyxia, especially asphyxia due to oxygen deficiency, autopsy findings are limited to the findings of acute death, namely dark red fluid blood, the presence of petechial hemorrhages, and congestion in various organs, with no findings specific to asphyxia. Molecular markers for the postmortem diagnosis of asphyxia have been reported, including proteins such as hypoxia-inducible transcription factor 1 alpha (HIF-1a), S100 calcium-binding protein B (S100B), ubiquitin C-terminal hydrolase L1 (UCHL1), stanniocalcin-2 (STC2), Cytochrome c (Cyto c), apoptosis inducing factor (AIF), nucleic acids (DNA and RNA) such as DUSP1, KCNJ2, miR-122, miR-3185, and metabolites such as lactic acid, pyruvic acid, and glycerol in brain, lung, other tissues, and body fluids (blood and urine) [11]. However, at present, few of those markers have been applied in actual forensic practice and expert testimony, and the development of new molecular markers that can be used as adjunctive diagnostics for asphyxia is a constant challenge.

Using animal models of CO_2_ intoxication and asphyxia due to oxygen deficiency, this study aimed to identify molecular markers with variable expression in the brainstem in each pathophysiological state and to develop molecular markers useful for differential diagnosis.

## Materials and methods

### Animals

Pathogen-free 8-week male C57BL/6 mice were obtained from Kyudo Co., Ltd. (Saga, Japan). All mice were bred and housed in a temperature-controlled (22 °C) environment with a 12 h light/dark cycle. The mice were fed with standard feed and given water *ad libitum*. All animal experiments were approved by the Animal Experimentation Committee of Kagoshima University (No. MD22003).

### Mouse model of CO2 intoxication and asphyxia due to oxygen deficiency

**Fig. 1:**
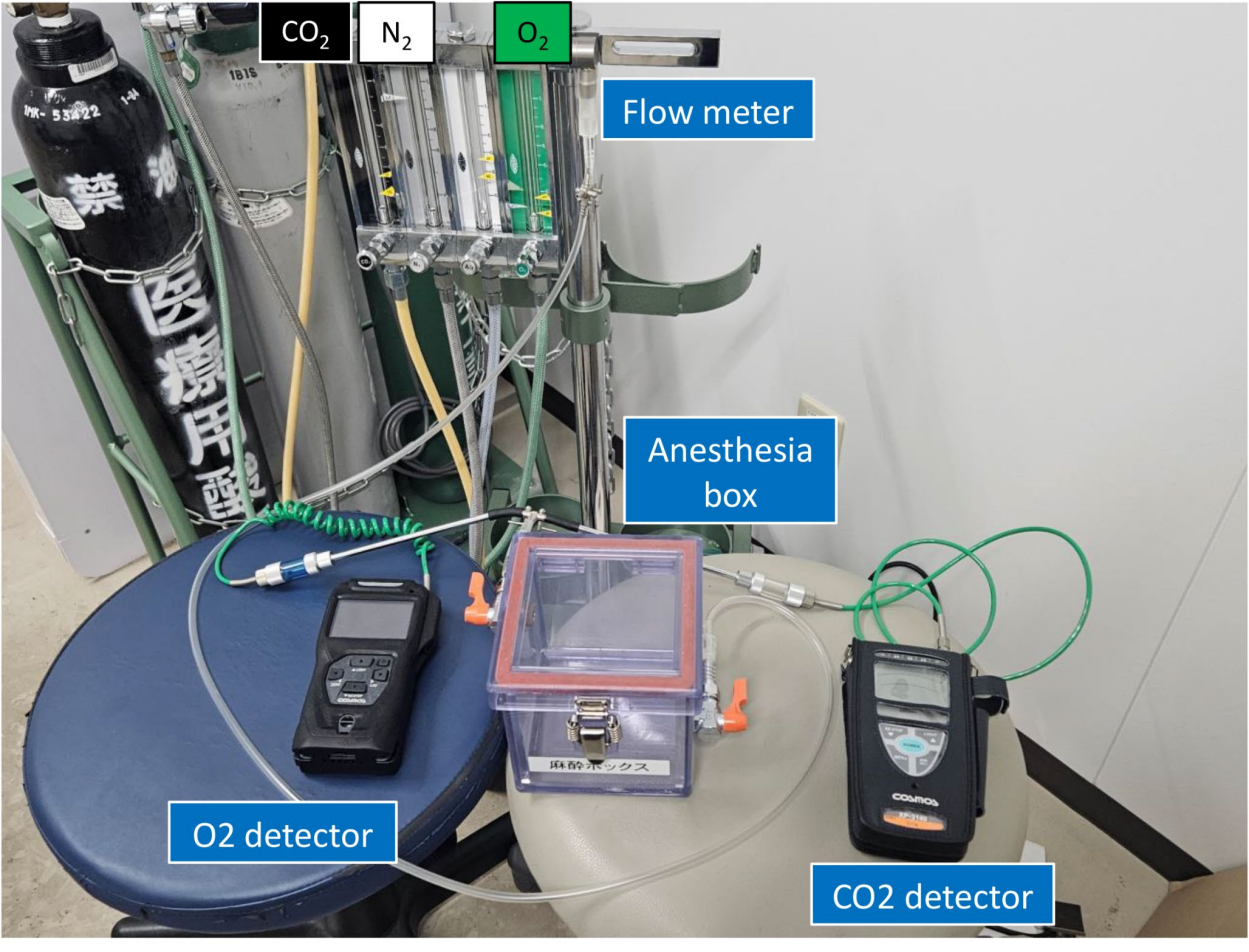
The experimental apparatus used to establish each model. The partial pressure of each gas in the box is regulated by a flow meter

The concentrations of CO_2_, O_2_, and nitrogen (N_2_) were adjusted using an experimental animal anesthesia device (Shinano Seisakusho, SN-487), and the attached anesthesia box was filled with the desired concentration of gas (Fig. 1). The mice were placed in the box and exposed to the gas. The gas concentration in the box was measured from the exhaust port of the box using an O_2_ concentration detector (New Cosmos Electric Co., Ltd., XP-3380II) and a CO_2_ concentration detector (New Cosmos Electric Co., Ltd., XP-3104), respectively. In order to examine the variation in gene expression, each model of CO_2_ intoxication and asphyxia due to oxygen deficiency was set at a gas concentration at which mice would survive for 10 to 30 min. The mice were divided into four groups: control (Group A), CO_2_ intoxication (Group B), high CO_2_/hypoxia (Group C), and hypoxia (Group D) (*n* = 3–5 in each group). Animals in Group A were euthanized by cervical dislocation under deep anesthesia. After confirming that the experimental mice had died, they were promptly removed from the box and the brains were removed. The brainstem section containing the respiratory center was immediately excised from the whole brain, immersed in RNAlater (Thermo Fisher Scientific K.K., Tokyo, Japan), and stored overnight at 4 °C. The RNAlater was then removed and the tissue was stored at -20 °C until RNA extraction.

### RNA-sequencing analyses

The RNA-sequencing analyses (RNA-Seq) were contracted to Bioengineering Lab. Co., Ltd. (Kanagawa, Japan) using the preserved brainstem section as a sample. Two libraries were generated for each of group by MGI Easy RNA Directional Library Prep Set (MGI Tech, Tokyo, Japan) and sequenced on a DNBSEQ-T7 (MGI Tech, Tokyo, Japan) with a read length of 2 × 150 bps. After normalization using the DEGES normalization method of TCC (ver. 1.34.0), edgeR (ver. 3.36.0) in an R/Bioconductor software package was used to identify genes with variable expression. The registered nucleotide sequences were compared with sequences in the KEGG database using BLASTN (ver. 2.13.0), and KEGG_IDs were assigned to the gene sequences (E-value < 1e-5). The up- and down-regulated genes were listed and each was subjected to Fisher’s exact establishment test.

### RNA isolation, cDNA synthesis, and quantitative real-time PCR

Total RNA was extracted from brainstem samples using Quick Gene RNA tissue kit S II (Kurabo Industries LTD., Osaka, Japan) and the quality of the RNA was assessed using DeNovix DS-11 (Scrum Inc., Tokyo, Japan). Complementary DNA (cDNA) was synthesized from total RNA (500 ng) using ReverTra Ace^®^ qPCR RT Master Mix with gDNA Remover Kit (Toyobo, Co., Ltd., Osaka, Japan) according to the manufacturer’s instructions. Quantitative real-time PCR (qRT-PCR) was performed using the StepOnePlus Real-Time PCR system (Thermo Fisher Scientific K.K., Tokyo, Japan). The reaction mixture consisted of a final volume of 25 µl containing 2 µl of cDNA sample, 10 µmol of a set of gene-specific TaqMan primers, 0.4 µmol TaqMan probe (TaKaRa Bio Inc., Siga, Japan), and 12.5 µl of Probe qPCR Mix (TaKaRa Bio Inc., Siga, Japan). We have designed gene-specific TaqMan primers and probes, as listed in Table 1, according to the result of RNA-Seq (Table 2). Cycling conditions included a denaturing step at 95˚C for 20 s, followed by 40 cycles at 95˚C for 1 s, and 60˚C for 20 s. The number of cycles of amplification required to reach the threshold (quantification cycle, Cq) were obtained using an amplification plot and the threshold line automatically reported. To determine the number of copies of the targeted mRNAs in the samples, the Cq values of the genes were normalized against that of Gapdh. The relative quantification of mRNA transcripts was performed using the ΔΔCt method.

**Table 1:** Primer and probe sequences for each gene used in qRT-PCR

Accession no.	Gene	Primer		TaqMan Probe
		Forward	Reverse	
NM_183022.3	Asic4	GGAAGTACAACCGCAATGAGAC	CCAGCAAGGTGAGGATGCTA	CCATGGAACAGCAAGCCGCC
NM_007913.5	Egr1	GAACAACCCTATGAGCACCTGA	AGAAGCGGCCAGTATAGGTGA	CAGCCAAACGACTCGGTTGCC
NM_022029.2	Nrgn	TCAACACCGGCAATGGACT	TCCAGCGGGATGTCAAGAATA	CGCCTGCTCCAAGCCAGACG
NM_013622.3	Oprd1	AACGTGCTCGTCATGTTTGG	CAATACTTGGCGCTCTGGA	TGGCTTTGGCTGATGCGCTG
NM_001379496	Sema3f	CTGCTCTCGCTACACAGCATC	TGCACAGACTCTACGGCATTC	AGTCGCCGGCAAGATGTCCG
NM_013697.5	Ttr	GAAGCCGTCACACAGATCCAC	CATCCAGGACTTTGACCATCAG	CCGCGGGTGCTGGAGAATCC
NM_173391.3	Tph2	GTATGGAGCAGGGTTACTTTCGTC	GGTCGTCTTTGGGTCAAAGG	CATGCTCTTTCCGACAAGGCGTG
NM_001289726	Gapdh	TGTGTCCGTCGTGGATCTGA	TTGCTGTTGAAGTCGCAGGAG	CCGCCTGGAGAAACCTGCCA

Asic4, Acid-sensing ion channel 4; Egr1, Early growth response protein 1; Nrgn, Neurogranin; Oprd1, Opioid receptor delta 1; Sema3f, Semaphorin 3f; Ttr, Transthyretin; Tph2, Tryptophan hydroxylase 2; Gapdh, Glyceraldehyde 3-phosphate dehydrogenase

**Table 2:** Number of genes with significant variation between each of the two groups in the RNA-Seq analyses

	A vs. B	A vs. C	A vs. D	B vs. C	B vs. D	C vs. D	Total
Number of genes	94	67	72	64	61	31	182

A, control group; B, CO2 intoxication group, C, combined group; D, hypoxia group

### Statistical analysis

The statistical significance of differences between means was assessed by one-way analysis of variance (ANOVA), followed by Tukey-Kramer’s test after confirming the normality of each data by Shapiro-Wilk test using EXCEL Statistics Ver. 8.0 (ESUMI Co., Ltd.). A *p*-value of < 0.05 was considered significant.

## Results

### Preliminary experiments for setting up each model

To establish models of CO_2_ intoxication, hypoxia, and a combination of high CO_2_ and low O_2_, we examined survival rates of mice at various CO_2_ and O_2_ partial pressures.

**Fig. 2:**
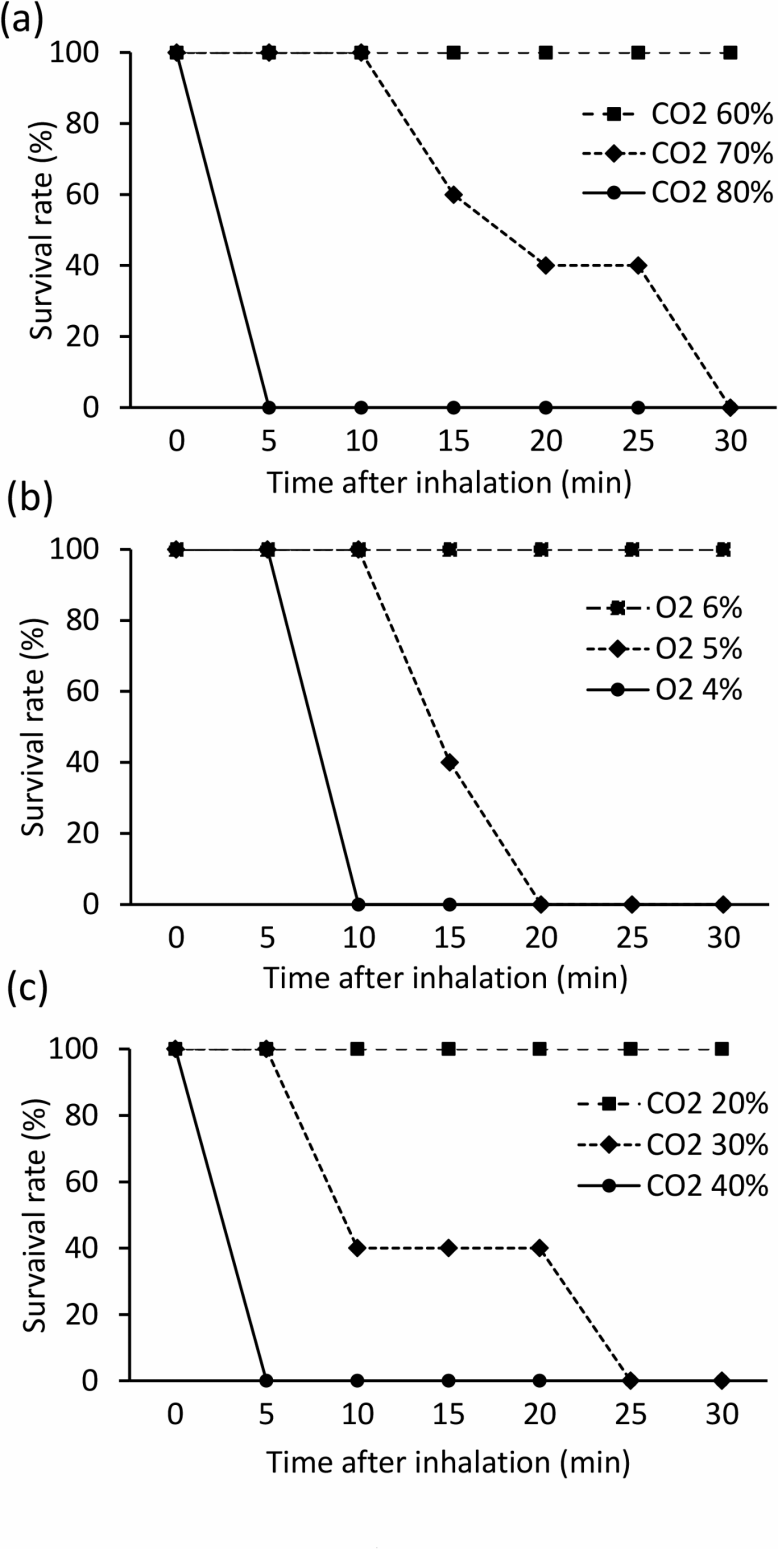
Survival rates in each model. (**a**) Survival rates at various CO_2_ partial pressures in the CO_2_ intoxication model. (**b**) Survival rates at various O_2_ partial pressures in the hypoxia model. (**c**) Survival rates at various CO_2_ partial pressures when the O_2_ partial pressure is set to 10% in the combined model

In the study of high CO_2_ concentrations, when the O_2_ concentration was 20% (Fig. 2a), all mice died within 5 min at 80% CO_2_ and between 10 and 30 min at 70% CO_2_. At 60% CO_2_, all mice survived for more than 30 min. In the study of low O_2_, when the CO_2_ concentration was 0% (Fig. 2b), all mice died within 10 min at 4% O_2_ concentration and between 10 and 20 min at 5% O_2_ concentration. At 6% O_2_ concentration, all mice survived for more than 30 min. Moreover, in the study of mixtures of high CO_2_ and low O_2_ concentrations (Fig. 2c), when the O_2_ concentration was 10%, all mice died within 5 min at 40% CO_2_ and between 5 and 25 min at 30% CO_2_. At 20% CO_2_, all mice survived for more than 30 min. From these preliminary experiments, the gas composition of each model was determined as follows: CO_2_ intoxication (Group B) at 70% CO_2_, 20% O_2_, and 10% N_2_ (mean survival time: 20.26 ± 0.97 min); combined (Group C) at 30% CO_2_, 10% O_2_, and 60% N_2_ (mean survival time: 13.3 ± 7.0 min); hypoxia (Group D) at 0% CO_2_, 5% O_2_, and 95% N_2_ (mean survival time: 14.25 ± 2.5 min).

### RNA-Seq

A total of 182 genes showed significant differences in expression between any two groups (Table 2). Of these genes, seven were selected based on the biological roles of their gene products as involved in CO2 intoxication and asphyxia due to oxygen deficiency: Acid-sensing ion channel 4 (Asic4), Early growth response protein 1 (Egr1), Neurogranin (Nrgn), Opioid receptor delta 1 (Oprd1), Semaphorin 3f (Sema3f), Transthyretin (Ttr), and Tryptophan hydroxylase 2 (Tph2). The results of the comparative expression analyses of these genes are shown in Table 3.

**Table 3:** The level of mRNA expression of each group of seven genes selected from RNA-Seq and comparison between groups

Gene (abbreviation)	Group				Significant difference (*p*-value)					
	A	B	C	D	A vs. B	A vs. C	A vs. D	B vs. C	B vs. D	C vs. D
Acid-sensing ion channel 4 (Asic4)	78	485	248	308	< 0.0001	< 0.0001	< 0.0001			
Early growth response protein 1 (Egr1)	396	595	776	813		< 0.0001	< 0.0001			
Neurogranin (Nrgn)	119	2122	808	1181	< 0.0001	< 0.0001	< 0.0001	< 0.0001		
Opioid receptor delta 1 (Oprd1)	136	48	107	116					0.0065	
Semaphorin 3 F (Sema3f)	105	400	214	259	0.00012					
Transthyretin (Ttr)	3414	537	147	8658	< 0.0001	< 0.0001	< 0.0001	< 0.0001	< 0.0001	< 0.0001
Tryptophan hydroxylase 2 (Tph2)	1902	1586	505	1947		< 0.0001		< 0.0001		< 0.0001

A, control group; B, CO2 intoxication group, C, combined group; D, hypoxia group

### qRT-PCR analyses

**Fig. 3:**
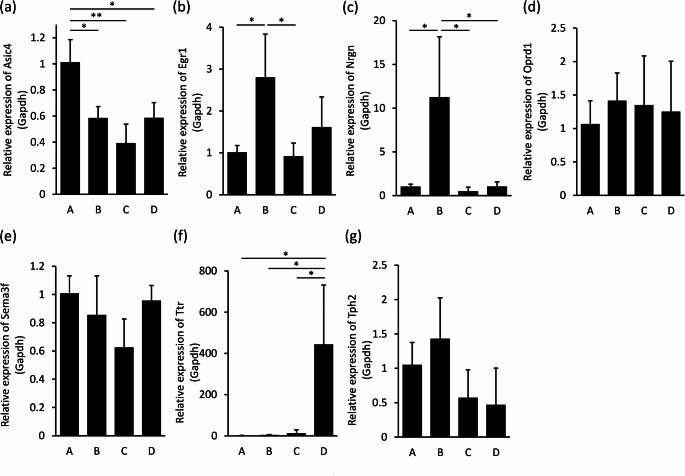
Relative expression of Asic4 (**a**), Egr1 (**b**), Nrgn (**c**), Oprd1 (**d**), Sema3f (**e**), Ttr (**f**), and Tph2 (**g**) in the brainstem in each group. Graphs show mean ± SEM. The statistical significance of differences between means was assessed by one-way ANOVA, followed by Tukey-Kramer’s test *p < 0.05, **p < 0.01

The mRNA expression of the seven genes selected by RNA-Seq were examined by qRT-PCR analyses. The expression levels of Asic4 were significantly reduced in CO_2_ intoxication (Group B), combined (Group C), and hypoxia (Group D) compared to Group A, but no significant differences were observed between the three groups (Fig. 3a). The expression level of Egr1 was significantly increased in Group B compared to Groups A and C, but there was no significant difference between Groups B and D (Fig. 3b). The expression level of Nrgn was significantly increased only in Group B compared to the other three groups (Fig. 3c). The expression level of Ttr showed a significant increase only in Group D compared to the other three groups (Fig. 3f). There were no significant differences in the expression of the remaining genes (Oprd1, Sema3f, and Tph2) among the four groups **(**Fig. 3d, e, g).

## Discussion

In the present study, we developed animal models to identify genetic markers for the differential diagnosis between CO_2_ intoxication and asphyxia due to oxygen deficiency. For Group D, the composition of the gas inhaled was 5% O_2_ and 95% N_2_. N_2_ is non-toxic and, like helium gas, is known to cause death by asphyxia due to oxygen deficiency when an enclosed space is filled with N_2_ [12]. In qRT-PCR analyses, Egr1 expression levels were significantly increased in Group B compared to Groups A and C, but there was no significant difference between Group B and Group D. Therefore, Egr1 is not a candidate for a specific diagnostic marker of CO_2_ intoxication. On the other hand, Nrgn expression levels were significantly increased only in Group B compared to the other three groups, suggesting that Nrgn may be a candidate diagnostic marker for CO_2_ intoxication. Since Ttr expression levels were significantly increased only in Group D compared to the other three groups, we consider Ttr to be a potential candidate for a specific diagnostic marker of hypoxia.

Neurogranin (Nrgn) is expressed abundantly in brain tissue and acts as “third messenger” substrate of protein kinase C (PKC)-mediated molecular cascades during synaptic development and remodeling [13]. The substrate, which is encoded by the Nrgn gene, binds calmodulin in the absence of calcium [14]. In the brain, Nrgn is expressed abundantly in the cerebral cortex, basal ganglia, hippocampus, thalamus, and hypothalamus, but is also expressed in the nuclei in the pons, such as lateral parabrachial nucleus, Kolliker-Fuse nucleus, and Solitary tract nucleus in the medulla oblongata, where the respiratory centers are located [15]. In recent years, Nrgn has been implicated in a variety of neurodegenerative diseases such as Alzheimer’s disease and Parkinson’s disease, psychiatric disorders such as schizophrenia and depression, and infectious diseases such as Creutzfeldt-Jakob disease, neuro-HIV, and neurosyphilis, and has been reported as a potential biomarker candidate for these conditions [16–20]. Among such studies, Hagihara et al. [18] reported that activity is increased, while working memory, prepulse inhibition, social behavior, and anxiety-like behavior are decreased in Nrgn knock-out (KO) mice compared to control mice, suggesting a schizophrenia model. They also observed increased brain lactate and lower brain pH in Nrgn KO mice. They hypothesized that the low brain pH is involved in the expression of various pre- and postsynaptic genes, including Nrgn, and causes schizophrenia-like symptoms. Therefore, we consider that the significantly increased Nrgn expression specific to the CO_2_ intoxication group (Group B) (Fig. 3c) is due to respiratory acidosis based on CO_2_ intoxication. On the other hand, there was no significant increase in Nrgn expression in Group C, despite the CO_2_ concentration (30%) being greater than that in the atmosphere (0.04%). As mentioned above, the induction of Nrgn expression may be caused by respiratory acidosis associated with increased CO_2_ concentration in the blood. In a previous study [21], there was a positive correlation between atmospheric CO_2_ concentration and the partial pressure of CO_2_ in the blood. The CO_2_ concentration in Group C (30% CO_2_) was lower than that in Group B (70% CO_2_), and it is thought that Nrgn expression did not increase because the concentration was not high enough to cause respiratory acidosis. Furthermore, even when the O_2_ concentration in the air is low, the CO_2_ concentration in the blood does not increase because CO_2_ is expelled as exhalation through breathing [22]. Therefore, we considered that there was no significant increase in the Nrgn expression in Group C.

Early growth response protein 1 (Egr1) is an immediate early gene (IEG) that regulates the transcription of various target genes and plays an important role in regulating responses to growth factors, DNA damage, and ischemia [23]. Egr1 is expressed widely in the brain, not only in the brainstem regions such as the midbrain, pons, and medulla oblongata, but also in the cerebral cortex, olfactory bulb, hippocampus, basal ganglia, thalamus, and hypothalamus [24]. In an in vitro model, Wen et al. [25] showed that Egr1 expression is increased in primary hippocampal neurons under hypoxic conditions. Egr family members, including Egr1, are upregulated in response to cellular stresses such as hypoxia and are involved in neuroprotection through the expression of target genes involved in cell growth, differentiation, and apoptosis. In our study, the significantly increased Egr1 expression in the CO_2_ intoxication group (Group B) compared to the control group (Group A) (Fig. 3b) may be due to respiratory center depression based on hypercarbonemia, resulting in cerebral hypoxia. In the hypoxia group (Group D), Egr1 expression levels did not increase significantly, but there was a trend toward an increase. Therefore, Egr1 cannot be a candidate as a postmortem diagnostic marker of CO_2_ intoxication, as it is thought to increase even in hypoxia.

Transthyretin (Ttr) is synthesized mainly by hepatocytes and epithelial cells of the choroid plexus (ChP), which are the sources of Ttr in plasma and cerebrospinal fluid, respectively [26]. Ttr is the carrier protein for thyroid hormones such as T3 and T4. Recent studies clarified that Ttr production by epithelial cells of the ChP is up-regulated to promote neuroprotection in the acute phase of ischemic stroke [27, 28]. Most studies using RT-PCR and in situ hybridization (ISH) indicate that Ttr is produced only in epithelial cells of the ChP in the brain and not in neurons and other resident brain cells. In our study, Ttr expression levels were significantly increased only in Group D, which may be a biological response to protect neurons from hypoxia. On the other hand, there was no significant increase in Ttr expression in Group C, despite the lower O_2_ concentration compared to the atmosphere. We assume that the hypoxia was not sufficient to cause a significant increase in Ttr expression in Group C, but was sufficient to increase Ttr expression significantly in Group D. In both humans and mice, the ChP consists of the left and right lateral ventricular ChP (LVChP), the third ventricular ChP (3rdChP), and the fourth ventricular ChP (4th ChP). Since the brainstem was used as the sample in this experiment, it is thought that Ttr production increased in epithelial cells of the 4th ChP contained in the brainstem. These results suggest that Ttr expression may be a candidate postmortem diagnostic marker for hypoxia.

Acid-sensing ion channel 4 (Asic4) is one of at least eight different Asic subunits (including Asic1a, 1b, 1b2, 2a, 2b, 3, 4, and 5) that are proton-gated voltage-independent ion channels [29]. Asics are expressed widely in the peripheral and central nervous system in both humans and mice. In the central nervous system, Asics are expressed in the pituitary gland most abundantly, but is also well expressed in brainstem regions, such as the pons and medulla oblongata, with roles in various important physiological functions and pathological states [30]. Although the role of Asic4 investigated in our study remains to be clarified, Asic1a has been found to exacerbate infarction by functioning to induce acid-induced neurotoxicity, i.e., neuronal cell death, during cerebral infarction [31]. Therefore, in the present study, the significant decrease in Asic4 expression in the experimental groups compared to controls may be a biological response to O_2_ deprivation in the brain. However, since Asic4 expression was decreased in all experimental groups compared with control, it is not a useful indicator for differentiating between CO_2_ intoxication and asphyxia due to oxygen deficiency, which was the main objective of this study.

Among other indicators we examined, Oprd1, which encodes a type of opioid receptor, and Sema3f, which encodes a receptor for the angiogenic factor Vascular endothelial growth factor, are both expressed in the central nervous system, and their expression has been reported to increase in response to hypoxia in the brain and to act in neuroprotection [32, 33]. Moreover, Tph2 encodes the rate-limiting enzyme for serotonin synthesis in the central nervous system and is expressed predominantly in serotonergic neurons in the raphé nucleus of the midbrain [34]. The elevation of CO_2_ concentration in the blood stimulates serotonergic nerves in the raphé nucleus, which in turn stimulates the motor nuclei of the diaphragmatic nerves in the spinal cord and promotes respiratory movement [35, 36]. However, contrary to our expectations, there was no significant variation in the expression of Oprd1, Sema3f, or Tph2 in experimental groups compared to control in this study. It is likely that the differences in O_2_ and CO_2_ concentrations and the time between inhalation of the gases and death prevented any significant variation.

Although few studies have investigated molecular diagnostic markers of CO_2_ intoxication, Sato et al. [37] reported that Alkylglycerone phosphate synthase (Agps) and Heat shock protein beta2 (Hspb2) expression in the frontal lobe and Pancreatic polypeptide (Ppy) and Corticotropin releasing hormone receptor 2 (Cchr2) expression in the hypothalamus were significantly increased in a rat model of CO_2_ intoxication and may be candidates for adjunctive diagnostic markers of CO_2_ intoxication. They concluded that the fluctuations in expression of those four molecules were caused by cognitive dysfunction and respiratory depression resulting from CO_2_ intoxication. Therefore, their research focuses on the symptoms of CO_2_ intoxication and targets the molecules involved. On the other hand, we focused on the pathogenesis of CO_2_ intoxication and used the brainstem region, where the respiratory center is located. Incidentally, neither Agps, Hspb2, Ppy, nor Crhr2 showed expression variation in their study, while, in this RNA-Seq study, we observed significant variation.

### Limitations

In this study, RNA was extracted from the entire brainstem and mRNA expression was examined (Bulk analysis). Cells that showed significant expression changes in Nrgn, Egr1, and Ttr were only discussed based on previous literature and were not identified here. We intend to consider single cell analysis in future studies.

Although we identified candidate genes as potential markers for postmortem diagnosis of CO_2_ intoxication and hypoxia using murine models, it is necessary to conduct similar studies using human autopsy samples to determine whether they can be applied to actual forensic diagnosis.

The composition of the gases used in this study was adjusted to allow survival for 10 to 30 min after exposure to search for gene expression that varies significantly with CO_2_ intoxication and asphyxia due to O_2_ deficiency. However, in actual cases, the gene expression changes found in this study may not be observed because death occurs in a shorter period of time when exposed to extremely high concentrations of CO_2_ or low concentrations of O_2_. Future studies should examine whether the increase in gene expression observed here is also observed in models of short-term mortality from exposure to even higher concentrations of CO_2_ or lower concentrations of O_2_. In addition, the changes in gene expression over time after exposure to the gases in the present model should be examined.

## Conclusions

In this study, we established a mouse model of CO_2_ intoxication and asphyxia due to oxygen deficiency. We showed that Nrgn mRNA expression was significantly increased in the brainstem of CO_2_ intoxication, and Ttr mRNA expression was significantly increased in the brainstem of asphyxia due to oxygen deficiency. Therefore, assessment of mRNAs of these genes in the brainstem may assist in the diagnosis/differential diagnosis of CO_2_ intoxication and asphyxia due to oxygen deficiency, respectively.

## Keypoints

1. We established mouse models of CO_2_ intoxication and asphyxia due to oxygen deficiency and investigated mRNA expression in the brainstem comprehensively.

2. We selected seven candidate genes identified using RNA-Seq.

3. Nrgn expression was increased specifically in the CO_2_ intoxication group, suggesting that the changes were due to respiratory acidosis caused by CO_2_ intoxication.

4. Ttr expression was increased specifically and markedly in the hypoxic group, possibly due to the protective effect of neurons from hypoxia.

5. Assays for mRNAs in the brainstem may assist in the diagnosis/differential diagnosis of CO_2_ intoxication and asphyxia due to oxygen deficiency, respectively.

## References

[1] La Verne AA, DiMaio DJ, Fernandez AJ. Occupational, accidental, explorational carbon dioxide inhalation poisonings, and prevention. PDM. 1973;4–5:83–94. 4802549

[2] Kuroki H, Yamazaki M, Nakamura M, Inoue H, Iino M, Honda K, Tuchihashi H, Matoba R. An autopsy case of CO_2_ (Carbon Dioxide) intoxication and the physiological mechanism. Jpn J Forensic Pathol. 2001;7:46–53. (In Japanese with English Abstract).

[3] Srisont S, Chirachariyavej T, Peonim AV. A carbon dioxide fatality from dry ice. J Forensic Sci. 2009;54:961–2. 19486434 10.1111/j.1556-4029.2009.01057.x

[4] Kettner M, Ramsthaler F, Juhnke C, Bux R, Schmidt P. A fatal case of CO_2_ intoxication in a fermentation tank. J Forensic Sci 20135;8:556–8. 10.1111/1556-4029.12058.12058 10.1111/1556-4029.12058 23316776

[5] Rupp WR, Thierauf A, Nadjem H, Vogt S. Suicide by carbon dioxide. Forensic Sci Int. 2013;231(1–3): e30-2. 10.1016/j.forsciint.2013.05.013 10.1016/j.forsciint.2013.05.013 23791381

[6] Murakami K, Kawaguchi T, Hashizume Y, Kitamura K, Okada M, Okumoto K, Sakamoto S, Ishida Y, Nosaka M, Kimura A, Takatsu A, Kondo T. Suicide by plastic bag suffocation combined with the mixture of citric acid and baking soda in an adolescent. Int J Legal Med. 2019;133:177–80. 29785586 10.1007/s00414-018-1856-y

[7] Yatsushiro M, Nakamae T, Katsuyama M, Miyamoto M, Hayashi T. A forensic autopsy case of suicide using baking soda and citric acid in a bathtub. Forensic Sci Med Pathol. 2024 Dec;6. 10.1007/s12024-024-00927-z. 10.1007/s12024-024-00927-z 39643789

[8] Takahashi M. Effects of carbon dioxide on human body. J Japan Soc Saf Eng. 1998;37:352–7.

[9] Gallagher KE, Smith DM, Mellen PF. Suicidal asphyxiation Byusing pure helium gas: case report, review, and discussion of the influence of the internet. Am J Forensic Med Pathol. 2003;24:361–3. 10.1097/01.paf.0000097856.31249.ac. 14634476 10.1097/01.paf.0000097856.31249.ac

[10] Gunnell D, Derges J, Chang SS, Biddle L. Searching for suicide methods: accessibility of information about helium as a method of suicide on the internet. Crisis. 2015;36:325–31. 10.1027/0227-5910/a000326. 26502782 10.1027/0227-5910/a000326

[11] Sacco MA, Aquila I. Post mortem molecular biomarkers of asphyxia: A literature review. Int J Mol Sci. 2024;25(21):11607. 10.3390/ijms252111607. 39519158 10.3390/ijms252111607 PMC11546465

[12] Giorgetti A, Pelletti G, Barone R, Garagnani M, Rossi F, Guadagnini G, Fais P, Pelotti S. Deaths related to nitrogen inhalation: analytical challenges. Forensic Sci Int. 2020;317:110548. 10.1016/j.forsciint.2020.110548. 33129047 10.1016/j.forsciint.2020.110548

[13] Xiang Y, Xin J, Le W, Yang Y. Neurogranin: a potential biomarker of neurological and mental diseases. Front Aging Neurosci. 2020. 10.3389/fnagi.2020.584743. 33132903 10.3389/fnagi.2020.584743 PMC7573493

[14] Jorgensen AN, Abdullah CS, Bhuiyan MS, Watt M, Dominic P, Kolluru GK, Kevil CG, Nam HW. Neurogranin regulates calcium-dependent cardiac hypertrophy. Exp Mol Pathol. 2022. 10.1016/j.yexmp.2022.104815. 35870494 10.1016/j.yexmp.2022.104815 PMC11118017

[15] Tissue expression of NRGN - The. Human Protein Atlas. https://www.proteinatlas.org/ENSG00000154146-NRGN/tissue/

[16] Davidsson P, Blennow K. Neurochemical dissection of synaptic pathology in Alzheimer’s disease. Int Psychogeriatr. 1998;10:11–23. 10.1017/S1041610298005110. 9629521 10.1017/s1041610298005110

[17] Koob AO, Shaked GM, Bender A, Bisquertt A, Rockenstein E, Masliah E. Neurogranin binds α-synuclein in the human superior Temporal cortex and interaction is decreased in Parkinson’s disease. Brain Res. 2014;1591:102–10. 10.1016/j.brainres.2014.10.013. 25446004 10.1016/j.brainres.2014.10.013 PMC4943923

[18] Hagihara H, Catts VS, Katayama Y, Shoji H, Takagi T, Huang FL, Nakao A, Mori Y, Huang KP, Ishii S, Graef IA, Nakayama KI, Shannon Weickert C, Miyakawa T. Decreased brain pH as a shared endophenotype of psychiatric disorders. Neuropsychopharmacology. 2018;43(3):459–68. 10.1038/npp.2017.167. 28776581 10.1038/npp.2017.167 PMC5770757

[19] Blennow K, Diaz-Lucena D, Zetterberg H, Villar-Pique A, Karch A, Vidal E, Hermann P, Schmitz M, Ferrer Abizanda I, Zerr I. Llorens FCSF neurogranin as a neuronal damage marker in CJD: A comparative study with AD. J Neurol Neurosurg Psychiatry. 2019;90:846–53. 10.1136/jnnp-2018-320155. 31097472 10.1136/jnnp-2018-320155

[20] Xiang Y, Xin J, Le W, Yang Y, Neurogranin. A potential biomarker of neurological and mental diseases. Front Aging Neurosci. 2020;12:584743. 10.3389/fnagi.2020.584743. 33132903 10.3389/fnagi.2020.584743 PMC7573493

[21] Fedde MR, Nelson PI, Kuhlmann WD. Ventilatory sensitivity to changes in inspired and arterial carbon dioxide partial pressures in the chicken. Poult Sci. 2002;81(6):869–76. 10.1093/ps/81.6.869. 12079055 10.1093/ps/81.6.869

[22] Ogasawara T. Pathophysiology of various oxygen-deprived conditions: medico-legal discussions from blood gas acid-base balance and blood flow values. J Tokyo Wom Med Univ. 1994;64(5):440–55.

[23] Xie Y, Li Y, Chen J, Ding H, Zhang X. Early growth response-1: key mediators of cell death and novel targets for cardiovascular disease therapy. Front Cardiovasc Med. 2023;10:1162662. 10.3389/fcvm.2023.1162662. 37057102 10.3389/fcvm.2023.1162662 PMC10086247

[24] EGR1 protein expression summary -. The Human Protein Atlas. https://www.proteinatlas.org/ENSG00000120738-EGR1/brain

[25] Wen J, Jiang Q, Yang L, Cui H. Transcriptomic hallmarks of hypoxic-ischemic brain injury: insights from an in vitro model. J Integr Neurosci. 2024;23(7):141. 10.31083/j.jin2307141. 39082286 10.31083/j.jin2307141

[26] Ueda M. Transthyretin: its function and amyloid formation. Neurochem Int. 2022. 10.1016/j.neuint.2022.105313. 35218869 10.1016/j.neuint.2022.105313

[27] Santos SD, Lambertsen KL, Clausen BH, Akinc A, Alvarez R, Finsen B et al. CSF transthyretin neuroprotection in a mouse model of brain ischemia. J Neurochem. 2010;115: 1434-44. pmid:21044072. 10.1111/j.1471-4159.2010.07047.x 21044072

[28] Talhada D, Gonçalves I, Gomes JC, Saraiva MJ, Reis Santos C, Ruscher K. Transthyretin expression in the postischemic brain. PLoS One. 2019;14(9): e0221555. 10.1371/journal.pone.0221555. eCollection 2019. 10.1371/journal.pone.0221555 PMC6719853 31479465

[29] Schwartz V, Friedrich K, Polleichtner G, Gründer S. Acid-sensing ion channel (ASIC) 4 predominantly localizes to an early endosome-related organelle upon heterologous expression. Sci Rep. 2015. 10.1038/srep18242. 26667795 10.1038/srep18242 PMC4678866

[30] Papalampropoulou-Tsiridou M, Shiers S, Wang F, Godin AG, Price TJ, De Koninck Y. Distribution of acid-sensing ion channel subunits in human sensory neurons contrasts with that in rodents. Brain Commun. 2022;4(6):fcac256. 10.1093/braincomms/fcac256. 36337346 10.1093/braincomms/fcac256 PMC9629378

[31] Storozhuk M, Cherninskyi A, Maximyuk O, Isaev D, Krishtal O. Acid-sensing ion channels: focus on physiological and some pathological roles in the brain. Curr Neuropharmacol. 2021;19(9):1570–89. 10.2174/1570159X19666210125151824. 33550975 10.2174/1570159X19666210125151824 PMC8762183

[32] Chung P, Chu Sin, Kieffer BL. Delta opioid receptors in brain function and diseases. Pharmacol Ther. 2013. 10.1016/j.pharmthera.2013.06.003. 10.1016/j.pharmthera.2013.06.003 PMC3775961 23764370

[33] Oinuma I. Signaling mechanism for axonal guidance factors, semaphorins. J Jpn Biochem Soc. 2015. 10.14952/SEIKAGAKU.2015.870428. [in Japanese with English Abstract]. 26571613

[34] Clark MS, McDevitt RA, Neumaier JF. Quantitative mapping of Tryptophan hydroxylase-2, 5-HT1A, 5-HT1B, and serotonin transporter expression across the anteroposterior axis of the rat dorsal and median Raphe nuclei. J Comp Neurol. 2006;498(5):611–23. 10.1002/cne.21073. 16917826 10.1002/cne.21073

[35] Wang W, Tiwari JK, Bradley SR, Zaykin RV, Richerson GB. Acidosis-stimulated neurons of the medullary Raphe are serotonergic. Neurophysiol. 2001;85(5):2224–35. 10.1152/jn.2001.85.5.2224. 10.1152/jn.2001.85.5.2224 11353037

[36] Depuy SD, Kanbar R, Coates MB, Stornetta RL, Guyenet PG. Control of breathing by Raphe obscurus serotonergic neurons in mice. J Neurosci. 2011;31(6):1981–90. 10.1523/JNEUROSCI.4639-10.2011. 21307236 10.1523/JNEUROSCI.4639-10.2011 PMC3071248

[37] Sato K, Tsuji A, Usumoto Y, Kudo K, Yokoyama T, Ikeda N. Expression of mRNA in the frontal cortex and hypothalamus in a rat model of acute carbon dioxide poisoning. Leg Med. 2016;19:101–6. 10.1016/j.legalmed.2015.07.014. 10.1016/j.legalmed.2015.07.014 26257316

